# Relation of Jump and Change of Direction Inter-Limb Asymmetries with Fitness in Youth Male Soccer Players

**DOI:** 10.3390/medicina59101749

**Published:** 2023-09-29

**Authors:** Yiannis Michailidis

**Affiliations:** Laboratory of Evaluation of Human Biological Performance, Department of Physical Education and Sports Science, Aristotle University of Thessaloniki, New Buildings of Laboratories, University Campus of Thermi, 57001 Thessaloniki, Greece; ioannimd@phed.auth.gr; Tel.: +30-23-1099-2233

**Keywords:** single-leg jump, 505 test, asymmetry, correlation, performance

## Abstract

*Background and Objectives*: Asymmetries between the lower limbs were evaluated by both researchers and practitioners as they seem to be related to the occurrence of injuries and the effect on performance. The purposes of this study were to (a) detect asymmetries of the lower limbs using a unilateral jump (single-leg countermovement jump (SLCMJ)) and 505 agility test, and (b) examine asymmetry effects on fitness (acceleration, speed, squat jump, Illinois agility test), in U16 youth male soccer players. *Materials and Methods*: Twenty U16 soccer players performed an SLCMJ and a 505 test to calculate the asymmetry index. They also performed the above fitness tests. The difference between the lower limbs was tested using the paired samples *t*-test (dominant vs. non-dominant). The correlations between the asymmetries of the lower extremities with the performance indicators were tested using the Pearson’s correlation test. The level of significance was set at *p* < 0.05. *Results*: The lower limbs differed significantly in the SLCMJ and 505 tests (*p* < 0.05). The only correlation was between the asymmetry in SLCMJ and performance in SJ (r = −0.641, *p* = 0.002). Asymmetries did not affect performance on most fitness tests. *Conclusions*: The two asymmetry tests identified asymmetries in different limbs. This indicates the variability in asymmetries and the need for specialized tests depending on the kinematic chain. The asymmetries observed should be eliminated using individualized training programs so that athletes are protected from injuries and from the possible negative impact on performance. Also, the detection of asymmetries in developmental age offers a greater time period for their elimination before the athlete enters professional sports.

## 1. Introduction

Soccer is an interval sport with periods of high, moderate and low intensity following each other. Of particular importance are the high-intensity actions with or without the ball, as they are the ones that characterize the performance of the players and their teams [1]. These actions are based on fitness and skills such as speed, power (jumping), agility and strength.

In soccer, as in other team ball sports, many actions are performed with one member. Soccer players exhibit a natural dominance in one leg, with the dominant leg being used for shooting, passing and dribbling, while the non-dominant leg serves mainly for support and balance. When these movements are repeated for years, they can lead to muscle asymmetries between the lower limbs [2]. Asymmetry is the difference in performance of the lower limb on specific tests [3]. In the literature, several ways of measuring the asymmetry of the lower extremities have been proposed. These can be divided into laboratory and field measurements. In the lab, asymmetry is measured using an isokinetic dynamometer at specific angular speeds [4], the back squat [5] and at specific angles of joints (unilateral mid-thigh pull) [6]. In the field, the unilateral jump tasks [7,8,9,10,11,12] and the unilateral change of direction (COD) tests [12] can be used.

Jumps are used very often as they are easy to apply and a large number of athletes (e.g., a soccer team) can be evaluated in a short period of time. For the detection of asymmetries, the unilateral countermovement jump, triple jump, standing broad jump and drop jump are the main jumps used. Regarding the use of jumps in a previous study, the use of a single-leg countermovement jump (SLCMJ) is suggested as it places greater emphasis on the production of force from one limb, which resembles several of the movements seen in team sports (e.g., jumps, sprints) [13].

As mentioned above, some COD tests can be used to detect asymmetries between the lower limbs. From the literature, it appears that the tests most commonly used for this purpose are the 505 test [5,12], the 180° turn test (a variant of the 505 test) [14] and the 90° cut COD test [5].

The evaluation of athletes and the finding of asymmetries among limbs have been carried out in recent decades with the aim of protecting them from injuries and improving their performance. A previous study mentioned [15] that soccer players with significant strength imbalances between their limbs had a higher risk of hamstring injuries. Additionally, altered biomechanics due to limb asymmetries can affect the distribution of forces on joints and increase the risk of overuse injuries. More specifically, Grindem et al. [16] and Nadler et al. [17] reported that asymmetries exceeding 15% increase the risk of injury. Even when an athlete returns to competitions after an injury, it is desirable not to show asymmetries higher than >10% [18,19]. Therefore, jumps are a sensitive and valid test to assess the asymmetry of the limbs that can be used both to prevent injuries and to return the athlete to play after injury.

In previous studies, an association between asymmetries and performance in fitness tests has been reported. Bishop et al. [7] observed that 12% of asymmetry in young female soccer players was associated with lower performance on the speed and jump test. In a recent study, Bishop et al. (2019) [20] observed that the asymmetries detected using the SLCMJ in elite U23, U18 and U16 soccer players negatively affected performance in jumping, speed and changing direction. Negative effects of lower limb asymmetry on tests of speed, jumping ability and change of direction in young soccer players were also observed in other studies [11,12]. Maloney et al. [21] observed in healthy adults that asymmetry was associated with lower performance on change of direction tests. In addition, the fastest participants showed a lower index of asymmetry.

However, there are also studies that found no effect of lower limb asymmetry on speed and agility. More specifically, Lockie et al. [9] studied the relationship between the asymmetry measured using the unilateral countermovement jump and performance on speed tests and change of direction tests in male students. Their results showed that an asymmetry of 10.4% did not affect performance on the other tests. In a more recent study, Dos’Santos et al. [8] observed no effect of asymmetry on the performance on two tests of change of direction in male students. The asymmetry was measured using the unilateral standing broad jump and was at 6%.

From the above, the effect of asymmetries on performance is not clear and the difference in results may be due to the different sample of studies (age, level), the different asymmetry assessment tests (horizontal or vertical jumps), as well as the relationship between asymmetry assessment tests and performance tests. Therefore, different studies are needed to control the above variables in combination to draw conclusions for specific populations. Thus, the purpose of the present study was to investigate the effect of lower limb asymmetries on the performance of developmental-age soccer players on fitness tests.

## 2. Methods

### 2.1. Participants

Power analysis based on previous studies was initially performed with an effect size of 0.6, a probability error of 0.05 and a power of 0.8 to find the minimum sample required for this study. From the results, it appeared that the minimum sample size was 19 participants and G*Power (Version 3.1.9.2, University of Dusseldorf, Düsseldorf, Germany) software [22] was used. Of the thirty-five players who said they would participate in this study, only 20 met the criteria and completed it (age: 16.6 ± 0.2 years; height: 176 ± 6 cm; weight: 71.1 ± 8.4 kg; percentage of body fat: 14.5 ± 2.6%). The criteria for participation were (a) not having musculoskeletal injuries for four months before the start of this study, (b) not being absent in more than 5% of training sessions and (c) not taking any kind of medication. All the players had more than 10 years of training experience in soccer. Parents signed consent forms after being informed along with participants about the study design. The local Institutional Review Board approved this study (Ap.N. 134/2023) in accordance with the Helsinki Declaration.

### 2.2. Study Design

The familiarization of the participants with the tests took place two weeks before the start of this study. During the first visit after the two weeks, the soccer players had their body mass, height and percentage of body fat measured. During the next 2 visits, the participants performed the fitness tests. The tests were performed at the same time of the day (6 p.m. to 8 p.m.). This study was carried out during the competitive season. Before each measurement, a 15 min warm up was performed and, at the end, a 10 min cool down. The players trained four times per week. One of them was a strength training session. Also, the participants consumed water ad libitum to ensure proper hydration during testing. 

The tests were applied by the same person in similar environmental conditions (~18 °C, ~30% humidity) on an artificial grass soccer field where every team had their training sessions.

### 2.3. Anthropometric Measurements

Body weight was measured with an accuracy of 0.1 kg, while height was measured with an accuracy of 0.1 cm (Seca 220e, Hamburg, Germany). The players wore only their underwear. A skinfold meter was used to measure body fat percentage and four skinfolds were measured (biceps, triceps, suprailiac, subscapular). Body fat was calculated using Siri’s equation [23]. The intra-class correlation coefficient for the measurement was 0.89. 

### 2.4. Speed Testing

The distance of 10 m was used to measure acceleration. For speed, although it has been reported that the distance needed by soccer players of developmental age U16 to develop the maximum speed of movement is between 30 and 40 m [24], in the present study, the distance of 30 m was used to compare the results with other studies. The players, from an upright position 0.3 m behind the starting line, ran as fast as they could over a distance of 30 m. Three photocell gates (Microgate, Bolzano, Italy) existed at 0, 10 m and 30 m [25], placed at hip height to avoid receiving incorrect signals from the movement of the limbs (hands) [26]. They performed two attempts, and the shortest time was used for statistics. The coefficient of variation for test–retest trials was 3.1 and 3.2%, respectively, for 10 m and 30 m.

### 2.5. Countermovement Jump (CMJ), Single-Leg Countermovement Jump (SLCMJ) and Squat Jump (SJ)

The jumps were performed by the players as described in a previous study [11]. For CMJ and SLCMJ, the participants were in an upright position with arms on hips and feet positioned hip-width apart. Their hands on all tests (CMJ, SLCMJ, and SJ) were placed on the waist. In CMJ, the soccer players from the above starting position would bend their knees at a 90° angle and jump as high as they could. In SLCMJ, they lifted one leg, and performed a countermovement to a self-selected depth followed by a quick upward vertical movement. In SJ, from a sitting position (knees at 90°), the soccer players jumped as high as they could without prior pretension (movement of the body downwards). Two attempts were made and the best performance was used for statistics. The electronic leap mat Chronojump of Boscosystem (Chronojump, Boscosystem, Barcelona, Spain) was used. If the technique of performing the jump was not appropriate, the examinee repeated it after one minute of recovery. The coefficients of variation for test–retest trials were 3.6% and 3.8% for Right SLCMJ and Left SLCMJ, respectively.

### 2.6. Illinois Agility Test

**Figure 1:**
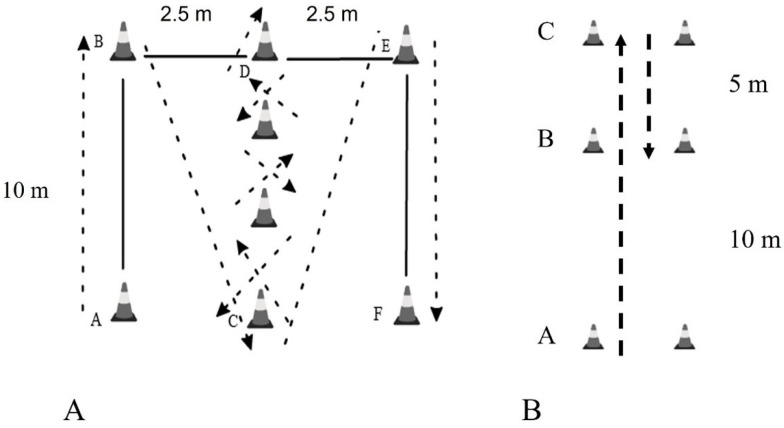
Description of (**A**) Illinois agility test and (**B**) 505 test.

The Illinois agility test was used to measure agility from an upright starting position according to the description of a previous study [27]. Soccer players from A sprinted to B and from there to C. They slalomed to D and returned in the same way to C. From there, they sprinted to E and from E to F. The distance was 60 m and photocell gates (Microgate, Bolzano, Italy) were placed at the start (A) and finish (F) line to record the time. They performed two attempts, and the shortest time was used for statistics. The test is shown in Figure 1A. 

### 2.7. 505 Test

This study also used the 505 agility test as described in previous studies [28] (Figure 1B). Participants from point A sprint for 15 m to point C where they make a 180° turn and sprint up to point B. According to the plan, four repetitions were performed, two turned with the right foot and two turned with the left foot. In any case, the best time was used. A photocell at the B point (line) was used to measure time (Microgate, Bolzano, Italy). The coefficients of variation for test–retest trials were 3.4% for the right leg and 3.6% for the left leg.

### 2.8. Asymmetry Index 

The index of asymmetry between limbs was calculated using the Jones and Bampouras (2010) equation [29] [(D leg − ND leg)/D leg] × 100. Also, in both the single-leg jump test (SLCMJ) and the agility test (505), the limb where the best performance took place was considered as dominant according to previous researchers [8].

### 2.9. Statistical Analysis

Initially, the normal distribution of our data was checked using the 1-sample Kolmogorov–Smirnov test and the results showed that we could use parametric statistical tests. Data are presented as means (±SD) and also, confidence intervals (CIs) (95%) were calculated. The intra-class correlation coefficient index (ICC) and coefficients of variation (CV) were used to analyze the reliability of the test. The magnitude for correlation coefficients was considered trivial (r < 0.1), small (r = 0.1–0.3), moderate (r = 0.3–0.5), large (r = 0.5–0.7), very large (r = 0.7–0.9), nearly perfect (r > 0.9) or perfect (r = 1.0) [30]. The paired *t*-test was used to test the differences between the two lower limbs, while the correlation of the asymmetry index with the performance was performed using the Pearson correlation test. The level of significance was set at *p <* 0.05. Note that asymmetries may favor either side depending on which limb scores larger [5]. The consistency of the asymmetries was calculated using the Kappa coefficient, and they were interpreted as poor (<0), mild (0.01–0.20), regular (0.21–0.40), moderate (0.41–0.60), substantial (0.61–0.80), almost perfect (0.81–0.99) and perfect [31]. The SPSS version 25.0 was used for all analyses (SPSS Inc., Chicago, IL, USA).

## 3. Results

**Figure 2:**
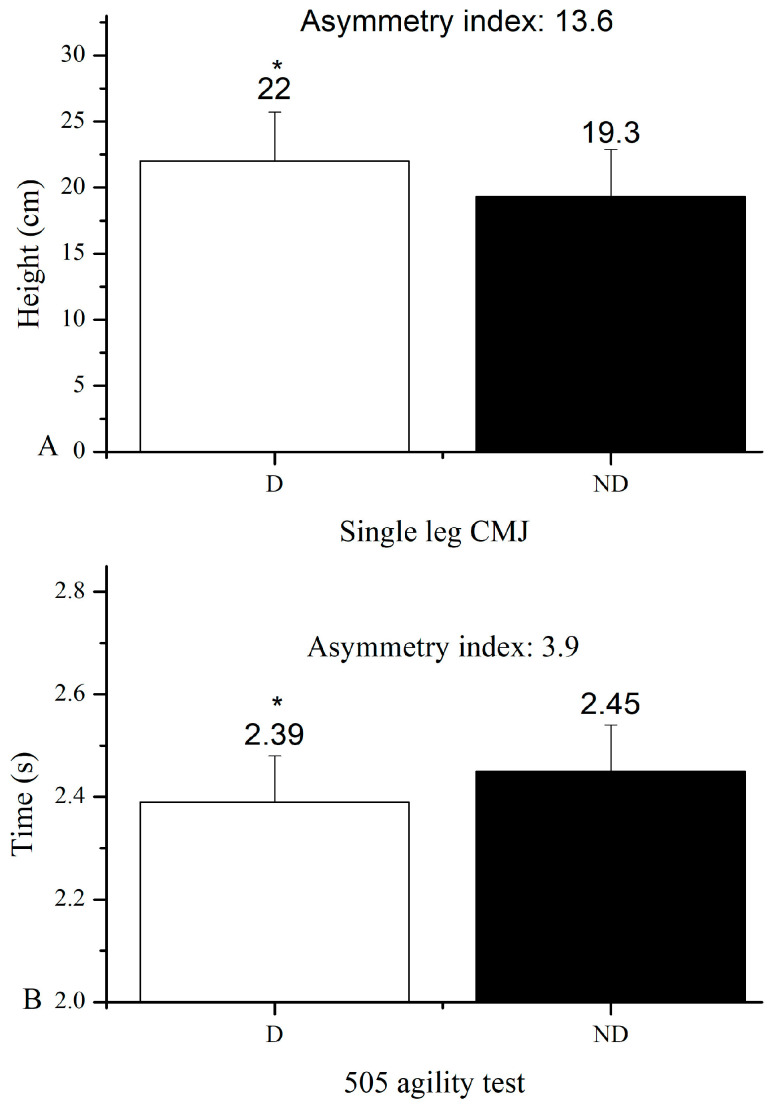
(**A**). Single-leg CMJ performance. (**B**) 505 agility test performance. D: dominant; ND: non-dominant; * denotes significant difference between D and ND lower limb.

As mentioned above, in all tests, confidence intervals were <10%, which made the test results acceptable. Figure 2 presents the asymmetry index from the two fitness tests between the dominant (D) and non-dominant (ND) limb (SLCMJ and 505 tests). The results showed a difference between the D and ND of the two limbs in the SLCMJ (t = 7.407, CI: 0.97–2.33, *p* < 0.001) and 505 tests (t = −5.089, CI: −0.13–0.05, *p* < 0.001).

The correlations between the asymmetries from the jump test and 505 tests are presented in Table 1. From the results, a large negative correlation was observed between asymmetry in jumps and performance in SJ (r = −0.641, *p* = 0.002). The level of agreement for the asymmetry scores (Kappa coefficient) between SLCMJ and 505 tests was k = −0.1 (none). Table 2 presents the performance of the players in the fitness tests and the statistical indexes of ICC and CV. 

## 4. Discussion

The aim of this research was to study the effect of asymmetries found using the SLCMJ and 505 tests on fitness tests in soccer players of developmental age U16. The results showed that the asymmetry between limbs found with SLCMJ was associated with performance only in SJ, showing a large negative correlation. The asymmetry index found with the 505 test was much smaller compared to that from SLCMJ (3.9 vs. 13.6, respectively). The asymmetry from the 505 test did not appear to be related to any of the fitness tests.

Our findings are consistent with previous studies. More specifically, a previous study observed that the use of different jump tests gives different indicators of asymmetry [4,6]. This fact indicates the specificity of the detection test with the movement evaluated. 

In a recent study [11], researchers tried to find the effect of lower extremity asymmetries on performance in acceleration and sprinting in two different age groups (U10 and U15) of developmental-age soccer players. The results showed no significant correlation between asymmetries and performance on the above physical ability tests (acceleration and speed). Also, in another study performed on sub-elite Qatar soccer players, the asymmetry rates observed were particularly low (2%) and did not seem to affect performance on fitness tests [32]. However, in a similar study [6], they tried to detect asymmetries using unilateral jumps and study their effect on speed and jumping ability. They used the SLCMJ on elite youth female soccer players and found that asymmetries affected time on speed tests. Additionally, another study from the same lab found that different jump tests to detect asymmetries had a different effect on performance. They observed that the asymmetry found with the drop jump was associated with performance in acceleration, speed and change of direction. However, no analogous effects on performance of the asymmetry index of SLCMJ were observed. In another study of the same laboratory [20] on elite U23, U18 and U16 soccer players, asymmetry appeared to affect their speed performance. Their study was conducted on female soccer players [33].

Regarding the ability to change direction [34], researchers observed little association between the non-dominant member SLCMJ and the L-run agility test. Moreover, the asymmetry in power (9.7%) found with the use of SLCMJ between the lower limbs did not differentiate the performance of athletes between dominant and non-dominant limbs. Michailidis et al. (2020) [12] in their study observed a correlation between lower limb asymmetry (SLCMJ was used) and performance on the 505 test in U15 soccer players, but not with the Arrowhead test. Bishop et al. (2019) [20] mentioned that male soccer players’ asymmetry reduces physical performance during the change of direction. However, in a recent study, Bishop et al. (2021) [5] studied the effect of the asymmetry displayed by the hop test on the performance on change of direction. This study was conducted on college-level athletes of various sports and it was observed that there were no correlations between asymmetries and performance on the COD test. Pardos-Mainer et al. (2021) [14] studied the effect of asymmetries in three different age groups (U18, U16, U14) of female soccer players on physical performance tests. The results showed that the asymmetries observed with the jump tests did not affect the performance of the COD tests. 

Regarding the use of the 505 test to check for the asymmetry in the change of direction between the two limbs, it was found that the differences were small. This finding is confirmed by other studies where the asymmetry index from the COD test was significantly lower than the corresponding index from SLCMJ [35]. The performance in the change of direction test depends, among other things, on technique. Therefore, when using it, we should know that we are not only evaluating factors related to fitness, but also technical factors, and only by using biomechanical analysis could differences be identified and quantified. Also, the lower specificity of the 505 test may be due to the fact that the one change of direction it includes is a small percentage of the total exercise time. As a result, any differences “disappear”. Perhaps the use of the direction test with multiple changes is more appropriate [36]. 

In the present study, a negative correlation was observed between the asymmetry found with the use of SLCMJ and performance in SJ. This finding is consistent with results from previous research. More specifically, Bishop et al. (2021) [7] observed a decrease in performance in the vertical jump as the asymmetry in SLCMJ increased in elite youth female soccer players. The same researchers also found that the asymmetries found with vertical jumping tests were not related to performance on horizontal jumping tests and vice versa. In another study of the same laboratory (Bishop et al. (2019)) [20] on elite U23, U18 and U16 soccer players, asymmetry appeared to negatively affect their jump performance.

The Kappa coefficient was calculated to see if the two asymmetry detection tests agreed on the dominant limb. The results showed that there was no agreement. Similar levels of agreement are reported in other studies [5,37,38] while greater agreement appears among tests that have similar motor patterns and based on similar physiological factors [14,37]. From the above, it is understood that asymmetry tests should be related to performance tests as they exhibit specificity. Therefore, when checking the asymmetry of an athlete, different tests should be used and will probably give different results.

Also, previous studies have reported that vertical unilateral jumps show higher asymmetry values compared to horizontal jumping tests [39,40]. This fact, as mentioned in the above studies, may be due to the more experiences children have in horizontal jumping activities compared to vertical ones. Furthermore, in the literature, it is reported that asymmetry has negative effects on performance when it exceeds 10% [7,9,39]. As mentioned above, the present study did partially confirm the above conclusion as asymmetry in SLCMJ was measured at 13.6% and was not correlated with sprint performance but only with SJ performance.

From the above, it is understood that the types of tests used to assess asymmetry affect performance on fitness tests. It was observed that the asymmetry from SLCMJ affects performance on the 505 test but not on the Arrowhead test [12]. Another study showed that the asymmetry from SLCMJ did not affect speed, while the asymmetry from the single-leg drop jump affected it [33]. Therefore, we must first select the type of movements that we want to test the asymmetry (e.g., vertical or horizontal jumping ability), and then select the test to carry out the assessment. Also, when the asymmetry exceeds 15%, personalized programs should be designed to eliminate this difference between the lower extremities.

In a previous study, it has been reported that in order for the test results to be reliable, the variability they show must be less than the differences between the two limbs [41]. In this study, limb asymmetries in jump tests were greater than CV values. Additionally, all CV values were <10%, which is considered acceptable [42].

The present study also has some limitations. The sample consisted of 20 developmental-age soccer players, so the findings of this study cannot be generalized to other populations (level, age, sport, gender). In addition, the fact that adolescents present specific characteristics (e.g., anthropometric) contributes to this. Also, the sample size does not allow separation according to playing position, a factor that can affect the results. Other parameters such as strength that can affect performance in tests such as sprints, jumps and changes of direction were not evaluated. In the present study, only two tests were used to detect asymmetry. As mentioned above, the use of multiple tests allows for better evaluation. Therefore, a larger sample should be used in future research. Also, because developmental characteristics can influence test results, age differentiation will enhance research results.

## 5. Conclusions

The SLCMJ and the 505 test can detect asymmetries between the lower limbs in soccer players. However, these asymmetries do not seem to affect performance in the speed and change of direction tests. The asymmetries shown using the SLCMJ were larger compared to those from the 505 test and different in the limb presented. This fact indicates the variability in the asymmetries and the need for specialized tests depending on the kinetic chain.

## Figures and Tables

**Table 1:** Correlations between variables.

Variable	SLCMJ Asym.			505 Test Asym.		
	r Value	*p* Value	CI	r Value	*p* Value	CI
0–10 m	−0.195	0.411	0.680–0.291	−0.049	0.838	−0.543–0.446
0–30 m	−0.134	0.574	−0.624–0.357	0.041	0.864	−0.454–0.536
SJ	−0.641	0.002 *	−1.021–−0.261	−0.072	0.764	−0.566–0.422
CMJ	−0.030	0.899	−0.525–0.465	0.250	0.288	−0.229–0.730
Illinois test	−0.196	0.407	−0.682–0.289	0.017	0.943	−0.478–0.512
505 test R	−0.340	0.142	−0.806–0.125	−0.037	0.877	−0.532–0.458
505 test L	−0.145	0.543	−0.635–0.345	0.072	0.762	−0.422–0.566
505 test asym.	0.419	0.066	−0.030–0.869			

* Denotes significant correlation.

**Table 2:** Results of fitness tests.

Test	Mean ± SD	ICC	CV (%)
0–10 m (s)	1.82 ± 0.07	0.86	3.1
0–30 m (s)	4.36 ± 0.13	0.88	3.2
Illinois agility test (s)	16.18 ± 0.57	0.79	3.6
SJ (cm)	35.9 ± 6.0	0.88	3.5
CMJ (cm)	39.4 ± 4.0	0.85	3.6

SJ: squat jump; CMJ: countermovement jump; ICC: intra-class correlation coefficient index; CV: coefficients of variation.

## Data Availability

The data presented in this study are available on request from the corresponding author. The data are not publicly available due to privacy restrictions.
